# Adjustable viscoelasticity allows for efficient collective cell migration

**DOI:** 10.1016/j.semcdb.2018.05.027

**Published:** 2019-09

**Authors:** Elias H. Barriga, Roberto Mayor

**Affiliations:** Department of Cell and Developmental Biology, University College London, WC1E 6BT, London, UK

**Keywords:** Collective migration, Viscoelasticity, Adherens junctions, EMT, Mechanical microenvironment, Cancer invasion

## Abstract

Cell migration is essential for a wide range of biological processes such as embryo morphogenesis, wound healing, regeneration, and also in pathological conditions, such as cancer. In such contexts, cells are required to migrate as individual entities or as highly coordinated collectives, both of which requiring cells to respond to molecular and mechanical cues from their environment. However, whilst the function of chemical cues in cell migration is comparatively well understood, the role of tissue mechanics on cell migration is just starting to be studied. Recent studies suggest that the dynamic tuning of the viscoelasticity within a migratory cluster of cells, and the adequate elastic properties of its surrounding tissues, are essential to allow efficient collective cell migration *in vivo*. In this review we focus on the role of viscoelasticity in the control of collective cell migration in various cellular systems, mentioning briefly some aspects of single cell migration. We aim to provide details on how viscoelasticity of collectively migrating groups of cells and their surroundings is adjusted to ensure correct morphogenesis, wound healing, and metastasis. Finally, we attempt to show that environmental viscoelasticity triggers molecular changes within migrating clusters and that these new molecular setups modify clusters’ viscoelasticity, ultimately allowing them to migrate across the challenging geometries of their microenvironment.

## 1 Introduction

### 1.1 Collective cell migration

Morphogenesis, wound healing, and cancer metastasis involve rearrangements of tissues which depend on the migration of large groups of cells. In order to efficiently migrate and reach their target positions, these groups of migrating cells synchronise their response to the environment and move in the same direction and at similar speeds [1,2]. A remarkable feature of migratory cell clusters is their high degree of directional motion as compared to individually migrating cells which, despite migrating with higher speeds than clusters, constantly change their direction of displacement [1,3]. This high order of directionality is a property that emerges from the exquisite coordination and communication that groups of cells achieve while migrating as a cluster. Such coordination requires cells not only to mechanically couple with their neighbours *via* fine regulation of junctional proteins, but it also involves fluent molecular communication among cells at different positions within the group, in addition to synchronisation of cytoskeletal activity [[1], [2], [3], [4], [5]]. This ensures that information from the microenvironment is transmitted from leader to follower cells– ultimately achieving a supracelullar polarised behaviour where the migration of a cluster resembles that of a single cell. This highly synchronised and cooperative mode of cellular motion is defined as collective cell migration [[1], [2], [3], [4], [5], [6], [7], [8], [9], [10]]

### 1.2 Models to study collective migration and their environments

Collective cell migration has fascinated scientists from diverse fields and this has driven the recent emergence of several model systems used to study *in vivo* and *in vitro* aspects of collective migration in various biological contexts [[1], [2], [3], [4], [5], [6]]. Collective cell migration can be observed all along an individual’s lifecycle, at early stages as it occurs during the migration of mesodermal cells during gastrulation in *Xenopus* [[11], [12], [13], [14]], laterality organ formation during zebrafish gastrulation [15,16], *Drosophila* epithelial cells migration during tracheal branching morphogenesis [17,18], *Drosophila* border cells migration [[19], [20], [21], [22]], collective directional migration of the *Xenopus* cephalic neural crest cells [[23], [24], [25], [26]], lateral line primordia migration in zebrafish [[27], [28], [29], [30]], *etc.* Collective migration is also observed at later stages during mouse mammary duct morphogenesis *in vivo* [31,32]. When adult tissues are repairing it is also possible to observe collective migration, sheets of epithelial cells need to migrate to close wounds [33,34] and when new blood vessels need to be formed during development or regeneration [[35], [36], [37]]. Although these cell types migrate across different micro-environmental contexts and use different strategies to maintain a coordinated and cohesively dynamic migratory group, the core regulators of the mechanisms that underlie these strategies are well-conserved [7].

**Fig. 1:**
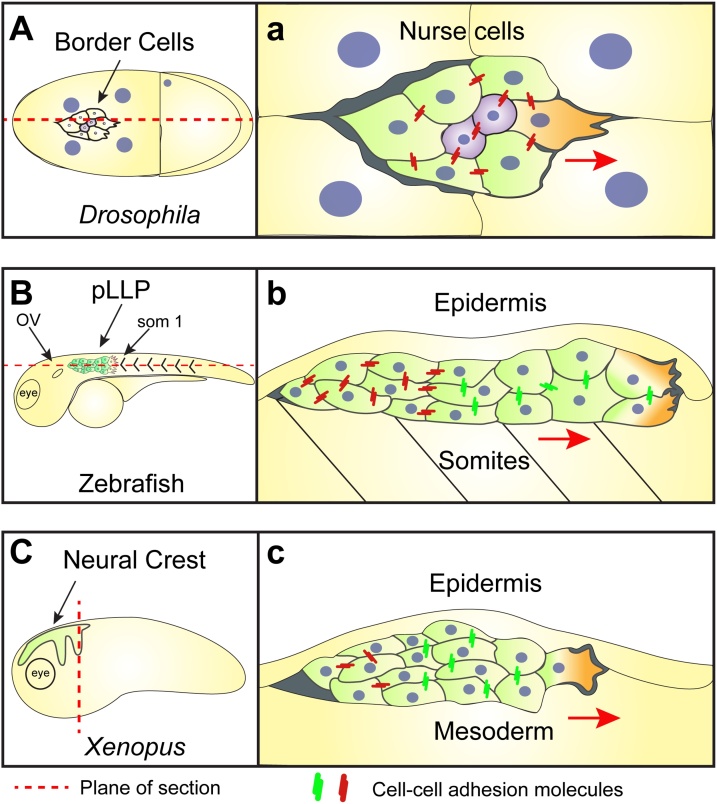
**Models to study collective cell migration***in vivo***and their microenvronments.** **A, a**. Schematics of *Drosophila* border cell anatomical position and collective migration. **(A)** Border cell migration in a *Drosophila* embryo. **(a)** Border cells migrate in a confined space, surrounded by gigantic nurse cells. High levels of AJs proteins are required to resist deformation and for the mechanical feedback require for directional collective migration. **B, b**. Diagrams showing topographic location and confined collective migration of the posterior lateral line primordia (pLLP). **(B)** Initial position of the pLLP among the otic vesicle (OV) and the first somite (som 1). **(b)** Sagittal section showing how the pLLP migrates in a confined space between the somatic mesoderm and epidermis. Differential antero-posterior distribution of AJs is observed, while E-cadherin localises to the rear, the leading edge express a more dynamic N-cadherin. **C, c**. Drawings representing anatomical position and confinement during neural crest collective migration. **(C)** In *Xenopus*, cephalic neural crest migrates as a collective from dorsal towards ventral and anterior territories in well-defined streams. **(c)** Diagram represent a transverse section across the head of a *Xenopu*s embryo and it shows the high degree of confinement experienced by the neural crest while migrating ‘sandwiched’ between the epidermis and underlying head mesoderm. Neural crest relies mostly in N-cadherin to mediate transient contacts require for the coordinated collective migration. Red arrows show the direction of migration for each tissue.

Here we provide details about the collective migration of *Drosophila* border cells, zebrafish lateral line primordia, and *Xenopus* cranial neural crest cells (Fig. 1A–C), as their migratory modes encompass most of the behaviours observed in other systems used to study collective migration. We also mention examples of collective cancer invasion. *Drosophila* border cells are a group of six to eight cells that originate from the follicular epithelium of the fly’s ovary and their migration is important for the proper morphogenesis of the micropyle, a structure required for sperm entry [19,20]. Border cells must first delaminate from the follicular epithelium before migrating as a collective towards the border between this tissue and the oocyte, position that confers these cells their name (Fig. 1A,a) [19,21,22]. While migrating, border cells are surrounded by gigantic nurse cells (Fig. 1a), in order to resist deformation to the stress that nurse cells exert on them and efficiently migrate in this confined space, border cells rely on mechanisms that allow them to dynamically maintain their shape, even while exchanging positions [38,39]. Zebrafish posterior lateral line primordia (pLLP) cells also migrate as a collective. pLLP migrates from anterior positions near the otic placode (Fig. 1B) until the caudal end of the embryo in a group of about 100 cells [[27], [28], [29], [30],^40^]. While migrating, pLLP deposit structures called neuromasts which are sensory organs that allow fish and aquatic amphibians to detect changes in the pattern of flow around their bodies [30]. pLLP migrates in a 3D microenvironment, confined between the epidermis and the mesoderm, with regulation of its migration involving mechanisms that confer it a less organised leading edge and a more organised posterior region (Fig. 1b) [27]. Another migratory cell population are the *Xenopus* cephalic neural crest cells, these cells are induced in dorsal territories between the neural and non-neural ectoderm (Fig. 1C), from where they migrate as a cell collective to form most of the vertebrate head [[23], [24], [25], [26]]. Before migrating, neural crest cells resemble a more epithelial phenotype, and in order to migrate must ‘lose’ their cell-cell adhesion strength *via* an epithelial-to-mesenchymal transition (EMT)-type process [24,25,41,42]. While migrating the neural crest experience high degree of confinement and in order to move it needs to open-up its way between the head mesoderm and the epidermis, and in addition must push the neural ectoderm in front of it (Fig. 1c). Consequently, the success of neural crest and all these collectively migrating cells in reaching their targets tissues relies on their ability to dynamically modify their migratory behaviour and, in doing so, adjust to this challenging environment.

Collective migration does not occur only in morphogenesis. Indeed, cancer cells often use strategies of collective migration which resemble that of the above discussed models [[43], [44], [45], [46]]. Some cancer cells can form multicellular streams, with cells displaying very loose adherens junctions but directionally migrating in the same path, in a ‘train-like’ motion but exhibiting higher persistence and slower speed than single cells [43,44,47]. This behaviour has been detected *in vivo* by using intravital multi-photon live imaging of human breast cancer cells grafted into adult mice, observing that multicellular streams, as opposed to individually migrating cells, correlate with a more invasive cancer phenotype [43,[45], [46], [47], [48], [49], [50]]. Another strategy used by cancer cells is collective invasion, a condition whereby cells migrate as highly coordinated collectives in which cells display a share higher degree of communication, directionality and speed of migration, as compared with stream-like migrating cells [7,[43], [44], [45], [46],^50^]. Collective invasion has been proposed to be used by several types of cancers, such squamous cell carcinomas, lung, and oral cancer [45,46,50]. Although some cancer cells use different strategies and can switch from collective to individual [51], recent studies revealed that collective invasive migration is one of the preferred strategies for cancer cells and that the collectiveness makes cancer cells more resistant to treatments, allowing for more efficient migration in 3D microenvironments [46]. Characterising the mechanical properties of native tumour microenvironments and their effect on the molecular and cellular behaviour of cancer cells may be crucial to generate more effective therapies. Mechanisms regulating border, neural crest, pLL, and cancer cells viscoelasticity during collective migration are discussed further in this review.

### 1.3 Physical challenges during cell migration

When migrating *in vivo*, embryonic, adult, and cancer cells must adjust their behaviour to interact with molecular and mechanical properties of their surroundings. Although the interaction among secreted molecules and their cognate receptors expressed by migrating cells have been largely studied [[52], [53], [54]], very little is known about the mechanical interaction among migrating cells and their surroundings [55]. The most common physical challenges that migratory cells experience *in vivo* are adhesion (friction), confinement, stiffness of migratory substrates, shear flow of extracellular fluids, topology and density of the surrounding tissues or extracellular matrix [56]. In order to confront these physical challenges of a 3D microenvironment, single cells rapidly modify their viscoelasticity to re-shape and ‘squeeze’ or to resist deformation [57,58]. However, in order to modulate their viscoelastic properties and confront their physical migratory microenvironment as a supracellular unit, cells within migratory clusters are required to coordinate the machinery that executes such changes. In this article, we review recent discoveries from the biomechanics field describing a range of mechanisms that individually migrating cells and clusters use to dynamically modify their viscoelasticity. We aim to illustrate that the fine regulation of viscoelastic properties of migrating clusters and those in their surroundings tissues are essential to ensure correct collective migration in varying contexts.

### 1.4 Viscoelastic behaviour of biological materials

**Fig. 2:**
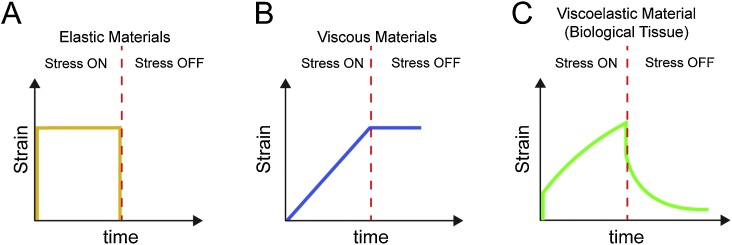
**Material response to mechanical deformation.** **A–C**. Drawings represent charts for the strain response of elastic, viscous, and viscoelastic materials to mechanical stress along time in hypothetical stress relaxation experiments. **A.** Elastic materials display proportional strain and stress. Elastic materials instantaneously strain upon stress and this deformation is fully and instantaneously reversed after removal of the applied stress. **B**. When a viscous material is stressed deformation is not instantaneous and it strain linearly with time in an irreversible manner (once applied stress is removed strain remains the same). **C**. Viscoelastic materials display time-dependent strain with an instantaneous elastic phase followed by time-dependent strain. Once stress is removed strain rapidly decrease as for elastic materials to then decrease, until some extent, in a time-dependent manner. Vertical dashed red lines indicate when mechanical stress is removed in the hypothetical stress relaxation experiment.

The vast majority of biological tissues have the ability to deform and adapt to their new environment when challenged by physical forces. Cells plated on 2D or 3D micro-patterned surfaces tend to adjust their shape to the one printed on the pre-patterned structure (reviewed in [59]). Also, when migrating *in vivo* cells tend to squeeze through extremely confined extracellular spaces and to adjust to these complicated geometries they largely deform (reviewed in [[59], [60], [61]]). Though the degree of deformation that cells or tissues display may vary depending on time or the type of tissue analysed, most biological tissues behave as non-linear viscoelastic materials when exposed to physical stress. A simplified definition for viscoelasticity of biological materials is that the same tissue exhibits viscous and elastic behaviours upon mechanical deformation [[62], [63], [64]] (Fig. 2A–C; for definitions of biophysical terms used in this article please see Box 1). Biological tissues have not always been studied as non-linear viscoelastic materials, pioneering research in mechanobiology would rather look to biological tissues as linear elastic materials, assuming Hookean behaviours when exposed to experimental deformation. However, in the second half of the 20th century, scientists started to observe that biological materials would exhibit elastic behaviours just when a temporal component was not being considered. At the time, several groups reported that, when considering time as an independent parameter in their experiments, the curves representing the response of biological tissue to experimental deformation would be rather non-linear, when compared to a control curve corresponding to a ‘Hookean material’ (for one of the first descriptions refer to [62]). This non-linear mechanical response of biological materials, has been recently re-visited and demonstrated by using purified and *in vitro* reconstituted cellular components– helping to explain why multiple biological material stiffen as they are strained in order to avoid large deformations and maintain tissue integrity [[65], [66], [67], [68]]. Further research and modelling work have lead physicists and biologists to generate a comprehensive framework where the response of biological materials to deformation is subdivided into a short-term linear elastic behaviour which is an instantaneous, almost time-independent response; and a time-dependent viscoelastic response which can be considered at various time scales and structural levels, such as short-term subcellular, mid-term cellular, and long-term supracellular regimes [63,[69], [70], [71]]. Altough short-term and mid-term regimes have been well-documented, observation of a long-term supracellular viscoelastic regime remains comparatively less studied [63]. While single cells can rapidly rearrange cytoskeletal components and reply to stress [72], clusters of cells may require more time when coordinately replying to environmental stress. Perhaps, due to the time delay imposed by the requirement of higher levels of coordination of these cytoskeletal rearrangements (proposed in [63]). In the subsequent paragraphs we provide examples of collectively migrating cell systems that respond to environmental challenges with a rather long-term viscoelastic regime. We discuss about cellular components involved in regulating the viscoelastic response of cells and tissues to deformation and some mechanisms regulating the dynamics of these cellular components.

Box 1 **Biomechanical terms glossary.**

**Viscosity**: The resistant of a fluid to flow easily explained by comparing water and syrup; water flows faster than syrup because it is less viscous, hence it opposes less resistance to flow.

**Elasticity**: The propensity of a material to return to their original position after a given load is removed. Elastic materials strain upon load and then load is removed they return to their original state. i.e.: elastic rubbers.

**Viscoelasticity**: A material that exhibit elastic and viscous characteristics when exposed to deformation is a viscoelastic material. Viscous or elastic response to deformation depends on the time scale. i.e.: when stress is removed the initial response is rapid and resembles the one of an elastic material, in a second phase the response will resemble more the one of a viscous material.

**Hookean behaviour**: Linear response to deformation observed in a perfectly elastic material. Stress applied to deform a material and the strain are equal.

**Stress**: Is use to express the load in terms of force applied to the cross-sectional area of a given object. It can also be defined as the force required to deform a material. A material can experience two types of stress, normal or shear stress. When a perpendicular force is exerted to an object it is experiencing **normal stress**, but if the force acts parallel to the surface of that object we talk about **shear stress**.

**Strain**: Deformations are normally observed when a material experiences stress and the ratio between its original shape and the deformation that the object suffered upon a given stress is the strain. As for the stress, a material can experience two types of strain, normal or shear strain. **Normal strain**: when a material is perpendicularly stressed. **Shear strain**: when stress acts parallel to the surface of a material. Blood flow on blood vessels for instance.

**Tension**: Is the reaction force applied by an object that has been stretched on the object that stretched it. Tension is a pulling force and as such it is active.

**Stiffness**: Is defined as the rigidity of an object or the extent of its resistant to deformation. The complementary property to stiffness is flexibility. To deform a stiff material, it takes higher force than to deform a soft material.

Alt-text: Box 1

## 2 Viscoelasticity of cellular components

In order to resist deformation or to deform without compromising the integrity of a cell or cluster, subcellular components are dynamically readjusted during cell migration. The machinery that allows cells to detect and respond to physical stress from their micro-environment involves sensing of mechanical inputs (mechanosensing) and their transduction into a biochemical response (mechanotranscduction) [73]. The cellular response to physical challenges triggered upon mechanical stress involves short- or mid-term adaptation of cell shape *via* cytoskeletal rearrangement, normally involving posttranslational modification of scaffolds, adhesion, polarity and contractility proteins [[74], [75], [76]]. In addition to this, a long-term response driven by modifications of gene expression profiles, that will then feedback into cell behaviour, may be also observed.

### 2.1 Classic mechanosensing

In adherent cells, sensing and transduction of mechanical inputs are complex processes and, herein, we briefly refer to a ‘canonical’ and simplified sensing mechanism (reviewed with more detail in [[77], [78], [79]]). One of the first structures proposed to be involved in mechanosensing are the focal adhesions points (FAs), where integrin adhesion receptors bind the cell to the extracellular matrix (ECM) [78,79]. This binding triggers the assembly of a group of proteins to form FAs that connect this integrin receptor with the cytoskeleton [76,[80], [81], [82], [83], [84]]. Ultimately, the force experienced at the FAs is transferred into the cytoskeleton by a protein complex that comprises, in part, vinculin and talin-mediated force transmission [81,[85], [86], [87]]. The cytoskeleton will then feedback into the focal adhesion to reinforce the dynamics of this structure *via* myosin-II-mediated contractility that will result in substrate deformation for cells migrating in compliant surfaces and cell deformation and migration for cells moving across stiffer substrata. Once deformed, the cytoskeleton will transfer these mechanical inputs from the environment into the nucleus to modify gene expression and biochemical signalling [84,88,89]. Sensing and transduction of environmental mechanical signals into a cellular and molecular response is not only mediated by the FAs-cytoskeleton feedback, mechanosensing also involve activation of mechanosensitive ion channels, such as Piezo1 [[90], [91], [92]], or mechanically responsive nuclear pores and the consequent activation of specific transcription factors [[93], [94], [95], [96]].

Although these are canonical mechanosensing strategies, the precise mechanism by which cells sense and respond mechanical inputs while migrating as a collective is just starting to be studied. *In vivo*, Fibronectin-based extracellular matrix of *Xenopus*, chick, or zebrafish embryos (Barriga and Mayor unpublished) has a thickness of about 10 μm and recent *in vitro* studies show that single cells could sense their substrate to a depth of about 15 μm [97], in this context a mechanism as the one described above perfectly fits with sensing of ECM by individually migrating cells. However, recent studies propose that collectively migrating cells exert 10-times larger forces into the substrate, hence the distance at which a migratory cluster of adherent cells sense physical changes on its micro-environment is deeper than the one registered for single cells [98]. However, how this larger sensing ability is achieved by the group or whether this is the case *in vivo* still need to be investigated.

### 2.2 Cytoskeleton

The cytoskeleton plays a major role in most of the mechanisms proposed to mediate sensing and transduction of mechanical cues from the micro-environment and its components undergo major modifications during cell deformation upon mechanical stress [99]. The building blocks of the cytoskeleton are biological polymers named intermediate filaments, actin filaments, and microtubules [100,101]. A balance in the rate of polymerisation/depolymerisation and degree of cross-linking of these structures determines whether a cell will deform or resist deformation upon mechanical stress. Rate of assembly and disassembly of these factors is controlled by an array of molecules. For example, actin nucleation factors initiate and extend polymer filaments, capping factors terminate filament growth and, on the other hand, depolymerising and severing factors disassembly these filaments [[101], [102], [103], [104], [105], [106], [107], [108]]. The mechanical behaviour and properties of these three filaments have been analysed in different systems, finding that microtubules represent the stiffer component of the cytoskeleton, with a less rigid network of actin filaments, and a rather soft network of intermediate filaments [99,100,[108], [109], [110], [111], [112]]. Mechanical strain of cytoskeletal network has also been measured in epithelial cells by using flow analysis, and mechanisms controlling cytoskeletal stiffening have been proposed [113,114]. Not only polymerisation and the passive mechanical properties of its components determine the mechanical state of the cytoskeleton, cross-linking factors have also been proposed to play a role in determining the architecture of cytoskeletal filaments and consequently their elastic state [[115], [116], [117], [118]]. The activity of molecular motors such myosin-II in combination with cross-linkers is also related to the viscoelastic properties of the cytoskeleton [89,119]. The interaction among filaments, motors, and cross-linkers is mechanically stimulated. For instance, myosin binding to actin fibres occurs in a force-dependent manner, as well as the contractile response of actomyosin to extracellular stiffness as shown by cell stretching and real-time imaging [72,88]. Actin assembly assays show that force-generating actin networks adapt to external mechanical force and that a force-feedback among intracellular and extracellular forces increases density and mechanical efficiency of newly branched actin networks [120]. Suggesting that a feedback between external and internal mechanical force could help migratory cells to adjust their viscoelastic properties and migrate in physically constrained microenvironments.

### 2.3 Nucleus

Intriguingly, not only the cytoskeleton and its components contribute to the viscoelasticity of cells. The nucleus has also been shown to be a limiting factor for the extent to which a cell would deform in order to migrate through confined spaces [60,121]. The nucleus is surrounded by nuclear membrane, underneath which exists a dense meshwork of proteins called nuclear lamina, the most abundant component of which are nuclear intermediate filaments formed mostly of proteins called Lamins [80,122]. Lamins are essential for nuclear-cytoskeletal coupling and mutations on these proteins impair the biochemical response of cells to mechanical stimuli [[123], [124], [125], [126], [127]]. Lamin has been shown to play a direct role in matrix-directed tissue differentiation. High matrix stiffness values given by collagen accumulation lead to high levels of Lamin-A and differentiation to bone, while low stiffness led to lower levels of Lamin and differentiation to fat tissue [128]. On the other hand, the nucleus plays an important role during cell migration in confined spaces by acting as a mechanical barrier that limits the degree of deformation that a migratory cell could experience. More specifically, the nuclear lamina is a limiting factor for nuclear deformability [60,124]. Whilst high content of Lamins act as a barrier for deformation, lower levels allow for a more flexible nucleus, however extremely low levels of Lamins limit cell survival [125,127,129]. One of the mechanisms controlling nuclear lamina density and nuclear viscoelastic properties has been recently described by using an elegant combination of microfabrication and quantitative biology methods. In this example a rapid accumulation of the actin nucleator Arp2/3 at the nuclear membrane of dendritic cells correlates with destabilization of the nuclear lamina, allowing cells to deform their nuclei [130], this may be relevant *in vivo* where cells need to modify their viscoelasticity while migrating across confined spaces.

### 2.4 Cell-cell junctions and emergence of coordination during collective migration

Recent advances are expanding our understanding of the role of cellular viscoelasticity during single cell migration, and how these properties can be adjusted by cells to efficiently migrate in confined spaces. However, during collective cell migration such mechanisms need to be coordinated to confer cells within the cluster with not only equal directionality and speed of migration, but also to allow the cluster to modify its viscoelasticity and confront physical challenges from the microenvironment as a supracellular unit. To coordinate these activities, cells within a migratory cluster need to mechanically couple and establish efficient channels of communication to transfer essential information from their environment across the group [131,132]. Cell-cell junctions play a key role in mediating these activities and fine adjustment of cell-cell adhesion strength has been shown to determine the fluidity of collectively migrating cells [133,134] and also their resistance to deformation when migrating across confined microenvironments [134]. Adherens and Gap junctions play a fundamental role in mediating cell-cell communication by allowing mechanical coupling, signalling and diffusion of information across channels. Both Adherens and Gap junctions respond to mechanical inputs. Myosin-dependent contractility of actin cytoskeleton organises and reinforces AJs at cell-cell contacts [135,136]. Gap junction activity forms part of the adhesion complex (adhesome) that coordinates intercellular forces during collective migration [137] and Connexins, the main component of Gap junctions, has been shown to be mechanosensitive proteins [138]. While cells can mechanically and molecularly couple *via* Adherens, Gap, and Tight junctions [139,140], here we refer mostly to Adherens junctions (AJs) as mediators of cell coordination and viscoelasticity during collective migration.

## 3 Tuning viscoelasticity during collective migration by regulating adherens junctions

An increasing body of evidence shows that the preferred mode of migration *in vivo* is collective cell migration. During collective migration, cells use Adherens Junctions (AJs) to mechanically couple and as an important source of signalling that coordinates collective behaviour. AJs play a crucial function in mediating the mechanical feedback among migratory cell clusters and their surroundings by coordinating force sensing and transmission across the cluster, and exertion of traction force into the migratory substrate [139,[141], [142], [143]]. Thus, clusters use different strategies to adjust their viscoelastic properties *via* fine-tuning of their AJs when migrating in their native environments (Fig. 2).

### 3.1 Cadherins and adherens junctions

One of the main components of AJs are proteins from the cadherin family. Cadherins are transmembrane glycoproteins containing an extracellular domain that mediates cell-cell adhesion *via* homophilic or heterophilic interaction and an intracellular domain that controls signalling cascades involved in a variety of cellular processes, including polarity, gene expression, *etc.* [136,[144], [145], [146], [147], [148]]. The spacing, strength of adhesion, and arrangement of AJs is context-dependent, with the content of cadherin depending on cell type. These proteins were initially described in cultured epithelial cells, where type-I cadherins, such as E-cadherin, provided cells with strong adhesions and its expression was regularly associated with static rather than motile phenotypes. Further analyses using *in vitro* and *in vivo* systems show that collectively migrating cells can also use type-I cadherins in their AJs to migrate as collective epithelia [31,32,51,149,150]. Recent advances revealed that collective migration is also possible for mesenchymal cells. For instance, N-cadherin-mediated AJs, allow neural crest cells within clusters to form transient and flexible contacts to migrate with high rate of neighbour exchange and fluidity, while maintaining collectiveness [151,152].

Depending on the context AJs can be highly dynamic or extremely stable and these dynamics can be modulated at each step of AJs formations. AJs forms in 3 main steps– first, cells contact each other in an initiation step where extracellular domains of cadherins are engaged, with the type of cadherin being expressed by contacting cells dictating the strength of this initiation step. A second step is the lateral expansion of a nascent adhesion, involving additional cadherin engagement to extend the area of contact. In a third step is the stabilization of the AJs, in which the cytoskeletal activity of collectively migrating cells are coordinated [25,142,148,151]. While the type of cadherin expressed by a given cell type may play a major role in the strength of adhesion at the initiation phase, turnover of cadherins is key to control the dynamics and duration of the lateral expansion and stabilization of AJs and, with that, the duration and strength of the contact. Cadherin content can be modulated at the transcriptional level by specific transcription factors as part of a process named epithelial-to-mesenchymal transition (EMT), and the turnover of these AJs proteins is modulated by posttranslational modifications.

### 3.2 Epithelial-to-mesenchymal transition (EMT) and adjustable viscoelasticity

**Fig. 3:**
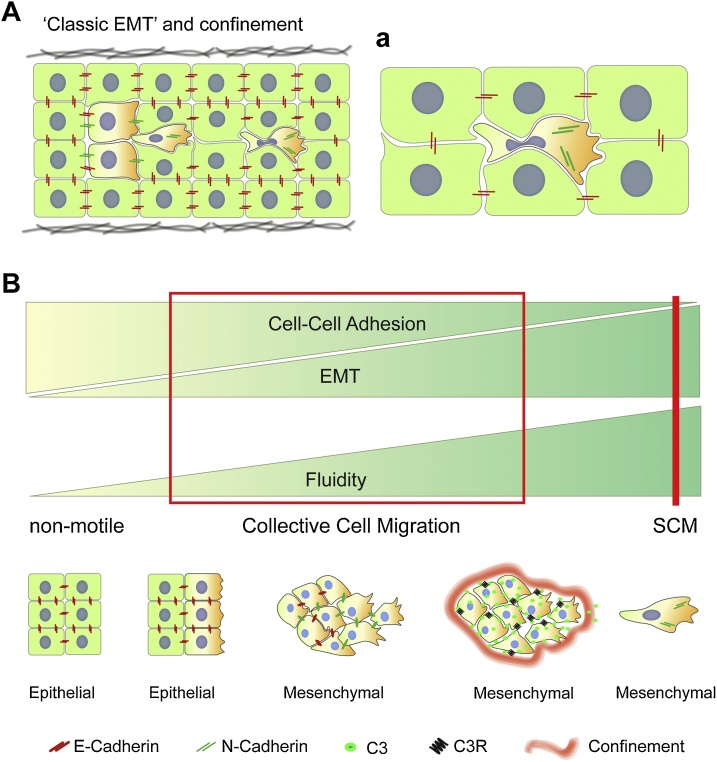
**EMT under confinement and optimal conditions for single and collective migration.** **A, a**. Classic model of linear EMT. **(A)** A sub-population of cells within an epithelia loss apicobasal polarity, undergos a switch in their cadherin content at AJs, became individual, and acquires mesenchymal migratory capabilities. When migrating *in vivo*, these cells are challenged by the physical strains of their surroundings. **(a)** The diagram shows the high degree of fluidity that a single cell reaches when migrating in confined spaces. Note that although the nucleus limits the degree of deformation that a cell can undergo, it can also deform to allow migration. **(B)** Schematic representation showing that for single cell migration (SCM) to occurs, cells require to acquire high degree of fluidity, which is very likely to be achieved by the extreme reduction in type-I AJs suffered during EMT. This EMT has to be highly regulated in order to maintain this low type-I cadherin levels and very importantly fluidity. Collective cell migration instead occurs just in optimal conditions of adhesion, fluidity, and EMT. In the absence of EMT an epithelial tissue has high strength of cell-cell adhesion and with that very low fluidity. If EMT is rather mild, this epithelium will maintain its epithelial behaviour with strong cell-cell adhesion mediated by type-I cadherins, this will help this tissue to resist deformation and open space to migrate in physically challenging environments (observed in wound healing or Drosophila border cell migration for instance). If the EMT rate increase, either by increasing the rate of cadherin turnover or using stronger transcriptional regulatory programs, cell clusters migrate as more fluid units that can deform in order to migrate in confined spaces (Neural crest or pLLP for instance). If the fluidity goes to a maximum, confinement and mutual attraction can help low adhesive forces to maintain collectiveness of the migratory group.

EMT is an initial step for single and collective migration and it was initially assumed as a process where cells from a static epithelium lose their apicobasal polarity and cell-cell adhesion properties to ultimately become single cells and migrate among tissues with a mesenchymal phenotype (Fig. 3A,a). At the level of AJs, a hallmark of this ‘canonical’ EMT is a strong reduction in the levels of type-I (E-cadherin) and, in some cases increase in type-II cadherins, or expression of type-I cadherins with reduced strength of adhesion, such N-cad [153]. Transcription factors from the Zeb, Snail, and Twist families are well-stablished as canonical upstream transcriptional regulators of this cadherin switch [[154], [155], [156]]. This definition was very informative at initial stages to unveil the basis of EMT, however most of this information arises from observations done in 2D *in vitro* experiments and does not account for the varying strategies that cells and groups of cells use to migrate across defiant *in vivo* environments. This field has evolved very fast and, consequent to new advances, EMT is no longer seen as a linear unidirectional process that confers motility to single cells but, instead, is viewed now as a more dynamic process that cells or groups of collectively migrating cells can use to adapt to physical challenges of their environment. It is well-stablished for example: that EMT is a reversible process and cells can also undergo MET (mesenchymal-to-epithelial transition) [157,158]; that EMT does not only promote motility but it also helps cells to maintain their stemness [159]; that migration can also occur in cells expressing high levels of E-cadherin [21]; and that EMT not only allows single cell migration, but it also contributes to collective epithelial and mesenchymal migration by conferring cells with adjustable viscoelasticity *via* modification of their AJs. This AJs modification occurs at a transcriptional and/or posttranslational level, by modulating expression and turnover dynamics of AJs proteins [25]. Several articles reviewing this new common framework for EMT have been published [[160], [161], [162]].

### 3.3 Tuning viscoelasticity in morphogenesis and wound healing

During morphogenesis, groups of cells migrate across confined spaces to give rise to increasingly complex forms. A clear example where cells tune their viscoelastic response at the transcriptional and posttranslational level during morphogenesis are the *Xenopus*’ cephalic neural crest. In order to move across the confined space between the epidermis and mesoderm these cells need to first switch their E-cadherin-based AJs to N-cadherin-based junctions. This process has been shown to be transcriptionally regulated by transcriptional repressor Twist1 in *Xenopus* [41] and by Sip1 in chick embryos [163]. Moreover, in order to move as a coordinated collective but in a fluid-like manner, the dynamics of N-cadherin-based contacts must be finely regulated. This regulation involves an increased rate of N-cadherin endocytosis in a mechanism that depends on the activity of the lysophosphatidic acid (LPA) and its receptor (LPAR2). Flow maps were used to show that this molecule was required to confer neural crest cells with low viscosity to allow flow into constrained spaces in the embryo [133]. Mechanical cues from the substrate have been recently shown to also control N- and E-cadherin protein levels in this and other systems [164,165], but whether this mechanical regulation occurs at the transcriptional or protein level remains unknown.

Another example is astrocytes, with these cells also migrating as a collective and their collectiveness relying on dynamic turnover of N-cadherin-based transient contacts. In these cells, a retrograde flow of N-cadherin is essential to reduce the strength of AJs and to maintain a fluid-like migration of the group. This retrograde flow involves N-cadherin internalization from the membrane, transit to the rear, and reutilization at the front of the cell where it can form new AJs. The mechanism controlling this cadherin tread-milling requires the polarized phosphorylation of p120-catenin [166,167].

Zebrafish blastodermal cells also offer a good example where cadherin turnover dynamics are controlled to modify the viscoelastic properties of a tissue. These cells do not undergo a switch of cadherins, but they do lose their apicobasal polarity and increase the turnover of E-cadherin to acquire a collective dynamic behavior. In this case, the endocytosis of E-cadheirn is enhanced by the transcription factor Pou5f1/Oct4 which activates the expression of EGF and promotes E-cadherin internalization [168].

Zebrafish lateral line primordia cells also regulate the strength of their AJs. These cells use both E- and N-cadherin to collectively migrate across confined spaces. Migrating primordia display a mesenchymal N-cadherin-rich leading edge and a more epithelial-like E-cadherin-rich rear, where the cells that will be deposited as neuromasts are located [[27], [28], [29]]]. Having a motile front and a static rear may help primordia to efficiently deposit neuromasts at the rear while maintaining directional migration at the front. Although this polarity in motility and cadherin content is clear [27,30], the precise mechanism that establishes cadherin polarity remain elusive– FGF and WNT pathways may play an important role in establishing this cadherin polarity [30].

While these examples show how cells modify their AJs to fine-tune their viscoelastic properties in order to acquire fluidity, other migratory clusters are required to ‘defeat’ their environment while collectively migrating. These clusters maintain high levels of type-I cadherins or even increase these levels to resist deformation from external mechanical stress and to exert higher traction forces while migrating. *Drosophila* border cells, for instance, need to resist the external pressure exerted by gigantic nurse cells and also open new spaces to migrate across them. To maintain a forward motion and to not collapse while moving across nurse cells, border cells increase their levels of E-cadherin as they delaminate and maintain these levels throughout migration [38,[169], [170], [171]].

Similarly, during wound healing, epithelial cells rely on E-cadherin-mediated AJs to migrate in a coordinated manner. In these cells, the expression of E-cadherin is maintained because it plays an important role in the transmission of traction forces generated by an actomyosin ring during migration [172,173]. Collective epithelial cell migration has been also observed in mammary branching morphogenesis, where cadherin-dependent tissue rearrangements allow for effective migration [31,174]. The activity of RAF and TGF-β has been shown to control tissue rearrangements and the rate of E-cadherin endocytosis in these cells [174,175].

Controlling cadherin endocytosis to regulate AJs dynamics has been proposed in many systems and is an incipient field of research for EMT and cell migration [146,[176], [177], [178], [179]]. The time that the mechanisms described here require to take place may contribute to the long-term regime of viscoelastic response to deformation proposed to act in collective migration [63]. Internalisation of cadherins occurs in the order of minutes [180] and transcriptional control can take even longer. Consequently, mechanisms of cadherins tuning may play a role in modulating this long-term viscoelastic regime.

### 3.4 Tuning viscoelasticity in cancer cells

Cancer cells also modify their viscoelastic properties to confront the environment *via* AJs adjustment during collective invasion. For this, cancer cells use a range of strategies, for instance during breast cancer invasion cells undergo EMT in a metalloprotease-dependent manner and then transcription factors will switch cadherins to reinforce EMT. In a first step, secreted MMP-3 metalloprotease cleaves the extracellular domain of E-cadherin leading to weakened cell-cell adhesion strength. Then, migration relies on Rac activation and the EMT transcription repressor Snail, which further reduces E-cadherin levels [181]. This allows collective migration to proceed in a fluid manner with less stringent cell-cell contacts, which are normally mediated by N-cadherin or L1CAM [[182], [183], [184]]. EMT is also reversible in cancer cells, where MET has been proposed to help mesenchymal circulating tumour cells to form secondary tumours [157,158]. In addition to this, inhibition of the EMT transcription factors Prrx1 [185]or Twist [186] can lead to MET activation. In this way, MET provides tumour cells with strong adhesion, enabling them to stop migrating, aggregate, proliferate, and become more aggressive.

In this context it appears clear that while single cell migration requires a maximum of fluidity and a minimum of cell-cell adhesion strength, collective migration occurs in an optimal condition where cell collectives can migrate as either monolayers of epithelial cells or highly dynamic mesenchymal clusters. These optimal conditions are microenvironment dependent and they emerge when cells find the right balance of cell-cell adhesions and fluidity in a mechanism which is mediated by a tuneable EMT program (Fig. 3B). *In vivo*, cell–cell adhesion strength is not always enough to maintain collectiveness of mesenchymal migratory groups and additional mechanisms help cells to collectively migrate (Fig. 3B). Mutual attraction and confinement have been shown to maintain collectiveness during neural crest collective mesenchymal migration. In neural crest cells mutual attraction is generated by chemotaxis towards C3a secreted by neural crest, which at the same time express the receptor C3aR [187]; confinement on the other hand is mediated, at least by the proteoglycan Versican [188]. The interplay of cell–cell adhesion tuning with other environmental and endogenous factors allow cells to push their strength of cell–cell adhesion to a minimum in order to acquire fluidity and migrate across extremely challenging conditions, without compromising collectiveness.

## 4 Environmental viscoelasticity triggers and direct collective migration

**Fig. 4:**
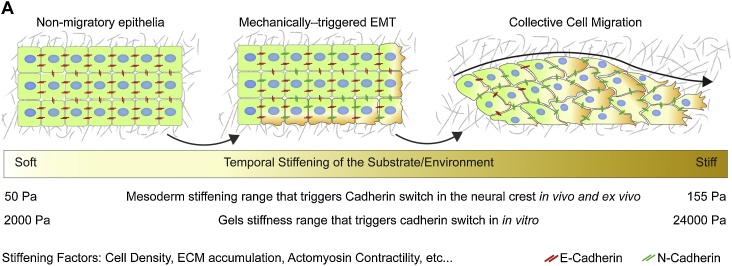
**Collective migration and AJs remodelling can be triggered by environmental mechanics.** **A.** The schematic shows how the mechanical interaction among migratory cell clusters and their surrounding tissues triggers AJs modification *via* cadherin switch. In this particular example, the tissue which is underneath a migratory cell population became stiffer as the developmental time progress. This stiffening is sensed by the migratory cell population which initiate an EMT-like program to modify their AJs in a switch from epithelial to mesenchymal type of cadherin, hence initiating fluid-like collective migration. This stiffening also provides an appropriate substrate for collective migration, where cells can efficiently polarise and form lamellipodia, required for their adherent migration. This mechanism was recently shown to trigger and support the collective migration of the *Xenopus* cephalic neural crest. This type of interactions may also operate in other systems where migratory substrate is observed. Stiffening *in vivo* has been shown to be mediated by, among other factors, actomyosin contractility, cell density, and ECM accumulation. Examples showing the elasticity ranges operating in the mechanical regulation of cadherins both, *in vivo* [165] and *in vitro* [228] are also shown.

Tissue viscoelasticity may represent a challenge for cells when they are already migrating. However, viscoelastic properties of the microenvironment can also play an instructive role in a wide range of processes which are just starting to be unveiled [[189], [190], [191], [192], [193], [194], [195]]. In migrating cells, viscoelastic properties of the substrate trigger the onset of collective migration and provide directional cues for migratory clusters. A large body of evidence shows that stiffer substrates allow adherent cells to acquire motility and provide them with an efficient migratory platform [[196], [197], [198], [199], [200], [201]]. Seminal research show that the strength of integrin to cytoskeleton adhesion of fibroblasts is dependent on matrix rigidity, with higher matrix stiffness leading to reinforced cell-substrate adhesion and stronger traction force generation in 2D gels [202,203]. Similarly, cancer cells show a more aggressive phenotype in stiffer microenvironments [43,44,204,205]. Fibrosis-enhanced metastasis is induced in cultured mammary gland tumour cells when its ECM is stiffened by increasing collagen cross-linking, in a mechanism mediated by the collagen cross-linker LOX [205]. Substrate stiffness also contributes to collective migration during wound healing. *In vitro* wound healing assays show that the migration of immortalised mammary epithelial cell monolayers is more efficient when plated on stiffer substrates, where they displayed faster propagation of forces and more coordinated migration [206]. The mechanism mediating this effect is a feedback loop between myosin-II contractility and the force-dependent reinforcement of cadherin-mediated AJs [206]. While *in vitro* studies show that viscoelastic properties of the substrate modulate cadherin content to allow efficient collective migration, this is only just starting to be analysed in more detail in living systems. In *Drosophila*, macrophages (haemocytes) migrate all over the fly embryo as single cells by transiently separating the interface between mesoderm and ectoderm. A recent article showed that the viscoelastic properties of their 3D environment are relevant for their invasive capabilities. This study shows that haemocyte invasion occurs in stereotypical locations, characterised by the presence of ectodermal cells with reduced cortical tension. These authors further demonstrate that tension in the ectoderm at this point was reduced in a mechanism that required the Tumour Necrosis Factor (TNF) activity [207]. This study demonstrated that, *in vivo*, single cell migration requires softening of the 3D environment. Another example occurs at late stages of development, during enteric system development. In this system, tissue viscoelasticity plays a dual role with an initial stiffening allowing neural crest-derivative cells to easily colonise the gut, however further stiffening reduces invasion rate. Collagen accumulation in the gut is the source of stiffening in this case and in order to keep migrating, cells constantly secrete proteases that help them degrade this dense matrix of collagen [192]. Viscoelastic properties of the migratory environment also play a role in triggering the onset of collective neural crest migration *in vivo* [165]. Stiffening of the mesoderm, its migratory substrate, occurs *via* cell accumulation underneath the neural crest in a PCP-dependent convergent extension movement of mesodermal cells, which starts early during gastrulation. Mesoderm stiffening triggered the onset of migration by promoting an EMT-like cadherin switch in neural crest cells, where N-cadherin was stabilised and E-cadherin levels reduced in stiff conditions. Once the onset of migration was triggered, stiffened substrate also provided an appropriate platform for neural crest cells to efficiently form actin-based protrusions which are required for their migration *in vivo* (Fig. 4A). An important gap in the field is the lack of *in vivo* measurements of tissue elasticity and fluidity/viscosity. *In viv*o measurements could deeply impact the relevance of our *ex vivo*/*in vitro* observations, by allowing the evaluation of cell behaviour and gene expression in a physiologically relevant context.

### 4.1 Environmental viscoelasticity and durotaxis

Viscoelastic properties of the environment not only trigger the onset of collective migration and provide a permissive environment for migration. These increasingly intriguing external cues have been also proposed to work as a directional cue during single and collective migration in a process called durotaxis, which is defined as the directional migration of cells along a stiffness gradient. *In vitro* experiments show that durotaxis occurs in single and collectively migrating cells. The direction of fibroblasts plated on polyacrylamide substrates can be biased towards pulling forces or against pushing forces exerted on the gels. This was one of the first demonstrations suggesting that changes in tissue rigidity and strain could play a role in guiding migration of cells [208]. Later attempts to characterise this durotactic behaviour showed that when single cells migrate from soft towards stiff surfaces their cytoskeleton and myosin phosphorylation profile became polarised, allowing for efficient durotaxis [209]. Collective durotaxis has also been observed *in vitro*. Monolayers of MCF10 A cells asymmetrically spread when plated on precisely designed stiffness gradients, with cells collectively spreading more efficiently towards stiffer parts of the gels. Cadherin-mediated AJs play a major role in the oriented transmission of forces that help cells to sense the gradient and achieve a global directional response [210]. Although these are *in vitro* studies, they have the potential to be relevant for morphogenesis, cancer, and wound healing. Viscoelastic properties from the environment also play an instructive role during vascular remodelling, in which endothelial cells migrate against the shear strain generated by the blood flow [[211], [212], [213]]. In one particular case, researchers observed that highly motile cells displayed increased mobile fraction of VE-cadherin (vascular endothelial E-Cadherin) at their AJs when migrating against the flow [213]. Altogether, these examples of mechano-environmental control of single and collective migration illustrate that the viscoelastic properties of the microenvironment are not just a barrier that need to be ‘defeated’ by cells in order to migrate, but instead are required to modulate different aspects of collective migration *in vivo*.

## 5 Interplay among viscoelasticity, EMT and the regulation of EMT transcription factors

**Fig. 5:**
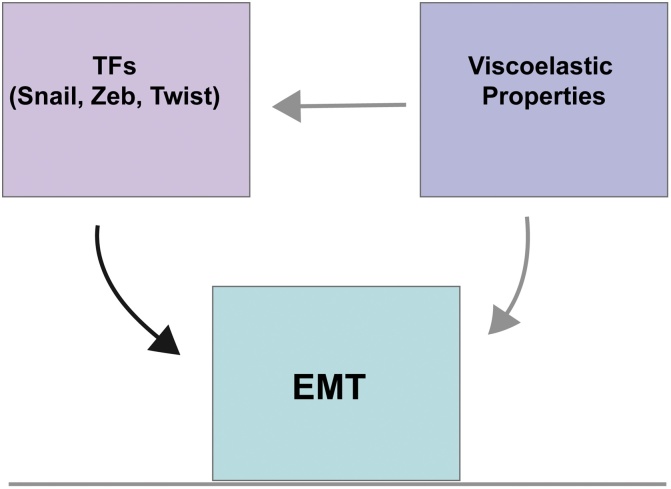
**Overview of the mechanical regulation of EMT and its transcription factors.** While the role of well-defined families of transcription factors in the initiation of EMT is well stablished, the role of mechanics in the regulation of these transcription factors and EMT itself is just starting to be elucidated. Based on new findings this is a promising area of research with great therapeutic potential in cancer and regeneration.

The extent of the effect of environmental viscoelasticity goes beyond cell behaviour, and several studies show that extrinsic mechanical cues lead to modification in gene expression. We have provided examples where AJs proteins are modified at transcriptional and posttranslational levels by a transient and reversible EMT, however the mechanisms underlying these modifications of cadherin content and the interplay of environmental mechanics with EMT-related transcription factors is just starting to be understood. Zeb, Twist, and Snail families of transcription factors are EMT transcriptional regulators that control the levels of Cadherin mRNA. Twist expression has been shown to be mechanically regulated during drosophila gastrulation, where external compressive forces induced ectopic expression of Twist [214,215]. Interestingly, the subcellular localization of Twist has been also observed to be mechanically regulated in 3D cultured MCF10 A and Eph4Ras cell lines. Twist nuclear localisation is observed when MCF10 A and Eph4Ras cells are cultured on stiff conditions, however in soft substrate Twist remains mostly in the cytosol [216]. The EMT transcriptional regulator Slug, from the Snail family, has been also proposed to be mechanically regulated. A vimentin-dependent mechanism of Slug mechanical activation has been proposed [217]. The expression of E47 (another EMT transcription factor) and SNAIL nuclear localisation and expression are also reduced when actomyosin contractility is inhibited [218,219]. The mechanism by which mechanical cues are internalised to control the expression of these transcription factors remains an open question. Yap1, a component of the hippo pathway, is a well-known transducer of mechanical inputs from the environment into biochemical responses [220,221]. Recent studies link Yap1 activity to EMT, mostly by interacting with EMT-transcription factors. The EMT transcription factor ZEB1 for example, became a transcriptional activator when interacting with YAP1 in aggressive types of cancer [222]. On the other hand, TGFβ interaction with SMAD proteins is essential for the transcriptional activation of SNAIL, SLUG, and TWIST. The stability of this TGFβ-SMAD complex has been recently shown to be mediated by Yap1 [223]. How Yap1 is internalised into the nucleus is just starting to be understood and recent advances show that force-dependent aperture of nuclear pores may be one of the main mechanisms of internalisation for Yap1 [95] and perhaps other transcription factors. These examples of interaction among mechanotransducers and EMT transcription factors illustrate one of the many possible mechanisms that migratory cells may use to transform mechanical inputs into a molecular and cellular response. This type of interactions may underlie other emergent properties of collective migration that rely in cell-cell interaction. For instance, it has been shown that cell-cell adhesion are required for collective Plithotaxis, a process which confer cell monolayers with ‘innate’ directionality [224]. While the role of these canonical transcription factors in the regulation of EMT is well-established *in vitro* and *in vivo*, the role of mechanical cues in the regulation of these transcription factors is comparatively less understood. Also, whether environmental viscoelasticity modulate EMT by controlling other aspects of this fascinating process such apicobasal polarity, cadherin turnover and MET is just starting to be unveiled (Fig. 5).

## 6 Concluding remarks and future perspectives

Here we discussed examples showing that the adjustment of the viscoelastic properties of migratory cells *via* EMT-regulated modification of AJs is essential to ensure efficient collective migration across tissues with physically challenging architectures. We also exposed that viscoelasticity of the migratory microenvironment is important to trigger cell migration, provide a substrate to allow efficient migration, and/or to guide migration *via* durotaxis. In addition, we examined new advances showing potential mechanisms mediating the translation of mechanical inputs into the cells, its effect on the expression of classic transcriptional regulators of EMT, and their effect on the modulation of the viscoelastic properties of migrating cells and their surroundings.

Examples of tissue interactions, such as the one showing that the EMT and collective migration of the neural crest are triggered by mesoderm stiffening *in vivo* [165], or the one showing that *Drosophila* haemocytes invade softer areas of the fly embryo [207], invite us to integrate our vision of morphogenesis as a mechano-molecular feedback loop that coordinate the timing of cellular rearrangements and gene expression profiles required for proper embryo formation. This integrated vision should consider that molecular signalling leads to cellular rearrangements that confer a tissue with new viscoelastic properties, and that this new viscoelasticity can now work as a long-range cue to modify the cellular, molecular, and viscoelastic properties of a neighbour tissue, in a sort of mechano-molecular feedback loop that coordinates morphogenesis. As tissue interactions during morphogenesis also occur at a chemical level *via* secreted molecules, it would be interesting to study the interplay of viscoelasticity and secreted molecules in the coordination of collective motion, as part of this integrative vision of morphogenesis. In the future, the consideration of these types of mechano-molecular feedback interactions may become particularly important in the improvement of protocols aiming to generate organ-analogous structures such organoids. So far, this field has focused mostly on the combination of the right molecular components, with viscoelastic properties of the microenvironment only just starting to be considered [225,226].

Furthermore, if we understand how these feedback loops operate in embryo morphogenesis we could consider applying this knowledge for more effective therapies when approaching major diseases such as cancer or when aiming for effective regenerative strategies. Using *in vivo* rat models, it was shown that mechanical stress exerted by gastric tumours into their microenvironment lead to major molecular effects which correlate with poor prognosis [227]. Characterising viscoelastic properties of wounds and regenerative tissues along with understanding how these modifications affect collective migration and cell fate, could also have an impact in therapies targeting these processes. Finally, viscoelasticity is a common property for the vast majority of biological materials and most of cells and tissues are under one or another physical force. Thus, the need of multidisciplinary approaches combining biophysical and biochemical parameters are key in order to reach a comprehensive understanding of increasingly complex biological systems.
